# AIDS-Related Pathology

**DOI:** 10.4061/2011/437431

**Published:** 2011-08-07

**Authors:** Liron Pantanowitz, Antonino Carbone, Justin Stebbing

**Affiliations:** ^1^Department of Pathology, University of Pittsburgh Medical Center, Pittsburgh, PA, USA; ^2^Division of Pathology, Centro di Riferimento Oncologico Aviano, Istituto Nazionale Tumori, Aviano, Italy; ^3^Charing Cross Hospital, Imperial College Healthcare NHS Trust, London, UK

Since the first announcement of acquired immune deficiency syndrome (AIDS) in 1981, around 33 million people worldwide are now infected with human immunodeficiency virus (HIV) [1]. This pandemic is associated with 6 million new annual HIV infections, resulting in 1.8 million deaths each year due to AIDS. During these past 30 years, an immense amount of knowledge regarding the pathology of HIV/AIDS has accrued. These valiant efforts have provided greater understanding about HIV/AIDS and its associated diseases and have undoubtedly improved the clinical management of afflicted persons. Despite this noteworthy effort, however, a tangible cure still seems remote as HIV infected individuals continue to succumb to many AIDS-defining and non-AIDS-defining conditions. In this special issue on AIDS-related pathology, we have assembled diverse papers that showcase this fascinating disease and underscore some of the many unanswered questions.

We are fortunate to have some truly superb papers with accompanying striking images. They teach us that the observation of subtle defects can immediately lead to diagnoses with enormous implications for the patient. Such conditions are often seldom observed outside of HIV. Occam's razor, the principle named after the 14th century philosopher William of Occam, does not apply in this setting. This generalization states that if there are a number of explanations for observed phenomena, the simplest explanation is preferred—called also scientific parsimony. Rare manifestations of common diseases and common presentations of rare conditions may be seen together; only HIV and its subsequent immunosuppression link these phenomena. Fortunately, with the advent of HAART in established market economies, the spectrum has changed with a notable shift from infection to cancer and “standard” diseases that non-HIV-infected people are routinely affected by, but often their manifestations have subtle differences.

A variety of diseases may be encountered related directly and/or indirectly to HIV infection. This is well illustrated in the paper reporting the spectrum of pathological manifestations of AIDS in a series of 236 autopsied cases in Mumbai, India. This article points out that the vast majority of underlying pathologies discovered by necropsy were either preventable or treatable conditions. The paper dealing with HIV-associated gastrointestinal (GI) disease diagnosed with endoscopic biopsy nicely portrays the variety of inflammatory, infectious and neoplastic diseases that may be seen in the upper and lower GI tract. Given the increased frequency of coinfections and broad diversity of diseases likely to be seen, it is not surprising that pathology specimens procured from HIV-infected are diagnostically challenging for anatomic pathologists. This is demonstrated in the beautifully illustrated article about multiple pathologies seen in skin biopsies from patients with HIV/AIDS, as well as the comprehensive review of HIV-related cytopathology.

Owing to the progressive reduction of the host immune system, HIV infected individuals are susceptible to many opportunistic infections. As the paper that stems from South Africa on primary oral tuberculosis shows, these often serve as an indicator for HIV infection. The article on *Penicillium marneffei* makes the point that in endemic areas like Hong Kong, disseminated penicilliosis is now included in the list of AIDS-indicator conditions. Human polyomavirus frequently reactivates in individuals infected with HIV, which typically manifests with sinister pathological consequences. This issue is addressed in the paper about novel human polyomavirus-associated cerebral disorders seen primarily in the era of highly active antiretroviral therapy (HAART). As HIV+ patients are increasingly qualifying for transplants, the complications of concomitant infection and management in this setting are manifold. The case report of zygomycosis associated with HIV infection and liver transplantation deals with just this conundrum.

The oncogenic viruses associated with HIV infection have incited much interest in the field over the years. Kaposi's sarcoma-associated herpesvirus/Human herpesvirus-8 (KSHV/HHV-8) was first found in 1994 by isolating DNA fragments of this virus from a Kaposi sarcoma (KS) tumor in an AIDS patient [2]. KSHV was subsequently found to cause several other diseases, such as primary effusion lymphoma and multicentric Castelman′s disease (MCD). The case report describing HHV-8 infection associated with KS, MCD, and plasmablastic microlymphoma in a single lymph node of a Kenyan man with AIDS commendably reviews the pathology of this oncogenic process. The number of reported cases of Epstein-Barr virus-(EBV) related smooth muscle tumor arising in patients with AIDS has been increasing since the mid 1990s. The epidemiology, clinical manifestations, pathologic features, prognosis, and management of this fascinating entity are methodically reviewed in this special edition.

HIV-infected patients are at increased risk of developing cancer. Despite the advent of HAART, malignancy in this population is a leading cause of morbidity and mortality. Like KS, AIDS-related non-Hodgkin lymphoma (AIDS-NHL) is one of the most common AIDS-defining malignancies. One of the articles in this edition is a five-year retrospective case series in which the authors review their institutions experience with numerous lymphoproliferative lesions in the setting of HIV infection. The pathological heterogeneity of AIDS-NHL reflects the heterogeneity of their associated molecular lesions [3]. Even though new molecular evidence continues to emerge regarding AIDS-NHL, several biological features about these AIDS-NHL remain perplexing. This is exemplified in the case report of a 47-year-old HIV+ man with a diffuse large B-cell lymphoma observed to undergo an immunophenotypic switch from a germinal center to nongerminal center origin. These authors offer several plausible explanations to explain this phenomenon.

The incidence and spectrum of non-AIDS-defining cancers (NADCs) continues to grow [4, 5]. As HIV-infected individuals live longer due to HAART, their risk of dying from one of these cancers is increased. The role of HIV-induced immunosuppression in the development of these NADCs appears to be less important than coinfection with certain viruses such as human papilloma virus (HPV). This edition offers an interesting review of the various carcinomas arising in the head and neck region in HIV+ patients, several of which are related to oncogenic viruses. While several NADCs are on the rise, it remains unclear why others such as breast cancers are decreased in this population [6]. The case report of an invasive ductal carcinoma of the breast surrounded by an intense lymphocytic response in the setting of HIV provides some insight into this interesting association, demonstrating the unique interplay between breast cancer and the HIV+ host's immune response. It is of great interest that the two hormonally driven tumors, breast and prostate cancers, may have a decreased incidence in the setting of HIV. Of course, many of the studies about HIV-associated breast and prostate cancer are small, and this may be a statistical aberration.

Apart from the direct and indirect effects related to HIV infection, treatment of HIV+ patients too has untoward side effects. For example, HAART may be associated with lipodystrophy, gynecoamastia, insulin resistance, hyperlipidemia and increased cardiovascular risk. Glitazones, a novel class of insulin-sensitizing antidiabetic agents, have been investigated in the management of HAART-associated lipid disorders. The effect of rosiglitazone, an agonist of peroxisome proliferator-activated receptor (PPAR**ƴ**), on skeletal muscle gene expression with regard to insulin sensitivity in individuals with HIV-insulin resistance is presented in this special AIDS-related edition of the journal. Additional basic science articles provide unique perspectives on osteoimmunopathology in HIV/AIDS and nanotherapeutics using a HIV-1 poly A and transactivator of the HIV-1 LTR-(TAR-) specific siRNA.

The challenges for diagnosis and management of sick people with HIV remains, despite the remarkable success of HAART. We should keep in mind that fewer than one million HIV-infected individuals are currently receiving antiretroviral therapy. Present anti-retroviral therapy costs between $10,000 and $20,000 per year, which provides excellent value for money in developed countries with a cost of about $10,000 per life-year saved; the lives saved are essentially the young and potentially economic productive section of the population. This compares very favorably with many other drugs for chronic therapies in current use. The limitations of anti-retroviral treatment strategies at a physical (suppression of viremia) and political (widespread availability) level have underscored the need to develop more effective strategies to control the spread and pathogenesis of HIV and the diseases related to it that we describe herein. In recent years, the demand for new antiviral strategies has increased markedly. There are many contributing factors to this increased demand, including the ever-increasing prevalence of chronic viral infections and other pathogens individuals are coinfected with, many of which are described in these papers.

While this great collection of papers certainly bears testimony to how much we have learned about HIV/AIDS to date, these papers also serve to remind us of how much we still do not understand. 

**Liron Pantanowitz** **Liron Pantanowitz** **Antonino Carbone** **Antonino Carbone** **Justin Stebbing** **Justin Stebbing**
